# The causal relationship between ever smoked and frozen shoulder: A two-sample Mendelian randomization

**DOI:** 10.1097/MD.0000000000035656

**Published:** 2023-11-03

**Authors:** Guang-hua Deng, Yong-kang Wei

**Affiliations:** a Ya’an Hospital of Traditional Chinese Medicine, Ya'an, China; b The Fourth Clinical Medical College of Xinjiang Medical University, Urumqi, China.

**Keywords:** ever smoked, frozen shoulder, Mendelian randomization

## Abstract

To investigate the causal relationship between ever smoked and frozen shoulder using a Mendelian randomization (MR) approach. Pooled data from a large-scale genome-wide association study were used. Genetic loci that were independent of each other and associated with ever smoked and frozen shoulder in populations of European ancestry were selected as instrumental variables. Inverse variance weighting was used as the primary analysis method. Weighted median and MR-Egger were used as complementary analysis methods to assess causal effects. To explore the causal relationship between ever smoked and frozen shoulder. Sensitivity test analysis was performed using heterogeneity test, multiple validity test, and leave-one-out analysis to explore the robustness of the results. Inverse variance weighting results of ever smoked showed an OR = 2.49, 95% CI = 1.05–5.91, *P* = .038, indicating that ever smoked is a risk factor for a frozen shoulder. And the test revealed no heterogeneity and pleiotropy, and the sensitivity analysis also showed robust results. This study used two-sample MR analysis to analyze and explore the genetic data, and the results showed a higher prevalence of frozen shoulder in patients with ever smoked, suggesting that active control of ever smoked may reduce the occurrence of frozen shoulder.

## 1. Introduction

Frozen shoulder, also known as adhesive capsulitis,^[1]^ is a pathological condition characterized by pain and limited joint motion in the shoulder.^[2]^ There are usually no significant findings in the patient’s history, clinical examination, or imaging evaluation to explain the loss of motion or pain.^[3,4]^ Ever smoked is a common risk behavior^[5,6]^ and has been found to contribute to the development and progression of many diseases.^[7,8]^ In recent years, several studies have investigated the relationship between ever smoked and frozen shoulder, but the findings have been mixed.^[9,10]^ Therefore, the causal relationship between ever smoked and periarthritis still needs further investigation.

In traditional epidemiological research methods, the causal inferences obtained are considered to be of limited value because of the influence of confounding factors and reverse causality.^[11]^ In contrast, Mendelian randomization (MR), a genetic epidemiological method, is a useful tool to assess the causal role of ever smoked and frozen shoulder.^[12]^ By using genetic variants such as single nucleotide polymorphism (SNP) as instrumental variables that can modify disease risk factors or exposures, MR studies can strengthen causal inferences about exposure-outcome associations.^[13]^ According to Mendel’s law of inheritance, genetic variants are not susceptible to confounding factors because they are randomly assigned during gamete formation.^[14]^ In addition, confounding factors and reverse causality correlations can be minimized because genotypes cannot change with disease progression.^[15]^

To this end, we conducted a two-sample MR study to examine the association of genetic susceptibility to ever smoked with frozen shoulder risk factors. We aimed to provide important evidence for the causal role of ever smoked in causing a frozen shoulder.

## 2. Data and methods

### 2.1. Data sources

The largest sample size of genome-wide association study (GWAS) data for ever smoked and frozen shoulder was obtained through the IEU OpenGWAS project (mr cieu. ac. uk). The website was accessed on 2023-06-06. The final population source for all data was Europe, of either sex. Including ever smoked (ieu-b-4858) containing 7,933,821 SNPs with a sample size of 99,996; frozen shoulder (ebi-a-GCST90000512) containing 15,184,371 SNPs with a sample size of 4,51,099. This study was a re-analysis of previously collected and published data and therefore did not require additional ethical approval.

### 2.2. Conditioning of SNP as an instrumental variable

First, the instrumental variables were highly correlated with exposure, with F > 10 as a strong correlation criterion.^[16]^ Secondly, the instrumental variable was not directly correlated with the outcome, but only influenced the outcome through exposure, meaning that there was no genetic pleiotropy. In this study, the MR-Egger regression model with a non-zero intercept term (*P* < .05) indicated the absence of genetic pleiotropy.^[17]^ Third, instrumental variables were not related to unmeasured confounding.^[18]^ Finally, the human genotype-phenotype association database Phenoscanner V2 was searched for phenotypes associated with instrumental variables at genome-wide significance levels to determine whether these SNPs were associated with potential risk factors.^[19]^

### 2.3. SNP screening

Significant SNPs were screened from the GWAS summary data of ever smoked (*P* < 5 × 10^−8^ was used as the screening condition)^[20]^; the linkage disequilibrium coefficient *r*^2^ was set to 0.001 and the width of the linkage disequilibrium region was 10,000 kb to ensure that each SNP was independent of each other.^[21]^ The above-screened ever smoked related SNPs were extracted from the GWAS summary data of frozen shoulder, while SNPs directly related to the outcome index were excluded (*P* < .05). The *F* value of each SNP was calculated, and SNPs with weak instrumental variables (*F* value less than 10) were excluded.^[22]^ And the human genotype-phenotype association database was queried to screen for potentially associated risk factor SNPs and to exclude them.^[23]^

### 2.4. Causality validation methods

The causal relationship between exposure (ever smoked) and outcome (frozen shoulder) was verified mainly using inverse variance weighting (IVW), supplemented by weighted median and MR-Egger MR analysis methods, using SNPs as instrumental variables.

### 2.5. Sensitivity analysis

Various methods were used for sensitivity analysis. First, the Cochran Q test was used to assess the heterogeneity among the individual SNP estimates, and a statistically significant Cochran Q test proved that the analysis was significantly heterogeneous. Second, the MR pleiotropy residual sum and outlier (MRPRESSO) was used to validate the results in the IVW model, correct for the effects of outliers, and if outliers existed, they were excluded and the analysis was repeated. Third, the MR-Egger intercept test was used to test the horizontal multiplicity of SNPs. If the intercept term in the MR-Egger intercept test analysis was statistically significant, it indicated that the MR analysis had significant horizontal multiplicity. Fourth, leave-one-out analyses were performed by removing a single SNP at a time to assess whether the variation drove the association between the exposure and outcome variables. Fifth, funnel plots and forest plots were constructed to visualize the results of sensitivity analyses. *P* < .05 suggests a potential causal relationship for MR analysis and is statistically significant. All statistical analyses were performed using the “TwoSampleMR” package in R software version 4.3.0.

## 3. Results

### 3.1. Instrumental variables

The current study screened 6 SNPs that were strongly associated with ever smoked (*P* < 5 × 10^−8^) without chain imbalance (*r*^2^ < 0.001, kb = 10,000). 6 SNPs remained by taking intersection with SNPs in the GWAS pooled data from frozen shoulder and also excluding SNPs directly associated with outcome indicators. In our study, each SNP had an *F* value greater than 10, indicating no weak instrumental variables (Table 1). We searched the human genotype-phenotype association database and no potentially relevant risk factor SNPs were found.

**Table 1:** Information on the final screening of ever smoked SNPs from GWAS data (n = 6).

ID	SNP	Effect_Allele	Other_Allele	β	SE	*P*	*F*
1	rs12731986	T	C	−0.0121	0.0022	2.17E-08	30
2	rs12874797	T	C	−0.0194	0.0034	1.06E-08	32
3	rs2011487	A	C	−0.0142	0.0021	3.96E-11	45
4	rs7829715	C	T	−0.0125	0.0021	4.12E-09	35
5	rs846184	T	C	0.0179	0.0032	1.54E-08	31
6	rs9287372	G	A	−0.0148	0.0021	4.05E-12	49

GWAS = genome-wide association study, SNP = single nucleotide polymorphism.

### 3.2. Causal relationship between ever smoked and frozen shoulder

**Figure 1.**
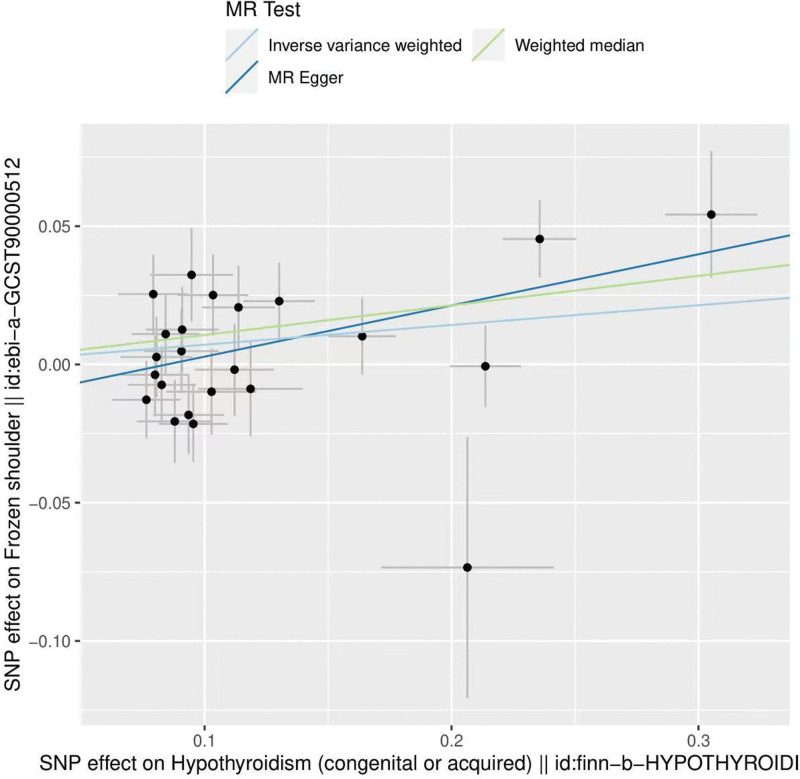
Scatter plot of ever smoked and frozen shoulder.

**Figure 2.**
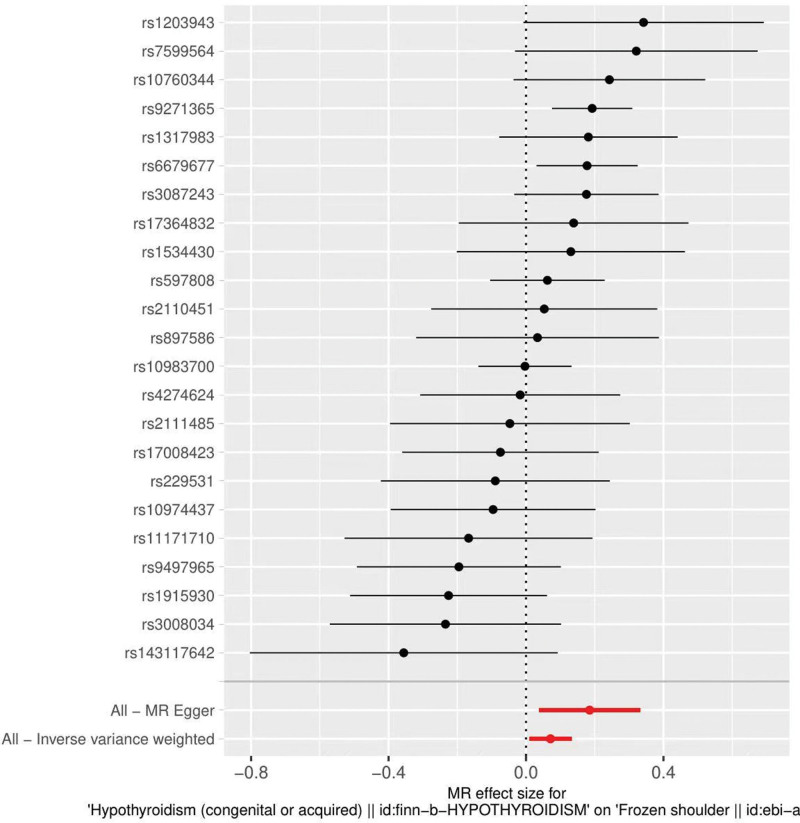
Forest plot of ever smoked and frozen shoulder.

The results of IVW showed a causal relationship between ever smoked and frozen shoulder. IVW: OR = 2.49, 95% CI = 1.05–5.91, *P* = .038. MR-Egger and weighted median show results in the same direction as IVW results (Table 2). We can see from both the scatter plot (Fig. 1) and the forest plot (Fig. 2) that ever smoked increases the risk of developing frozen shoulder.

**Table 2:** MR regression results of the 3 methods.

Method	β	SE	OR (95% CI)	*P*
IVW	0.912	0.441	2.49 (1.05–5.91)	.039
WME	1.011	0.558	2.75 (0.92–8.20)	.070
MR-Egger	3.708	2.581	440.77 (0.15–10902.67)	.263

IVW = inverse variance weighting, MR = Mendelian randomization, WME = Weighted median.

### 3.3. Sensitivity analysis

**Figure 3.**
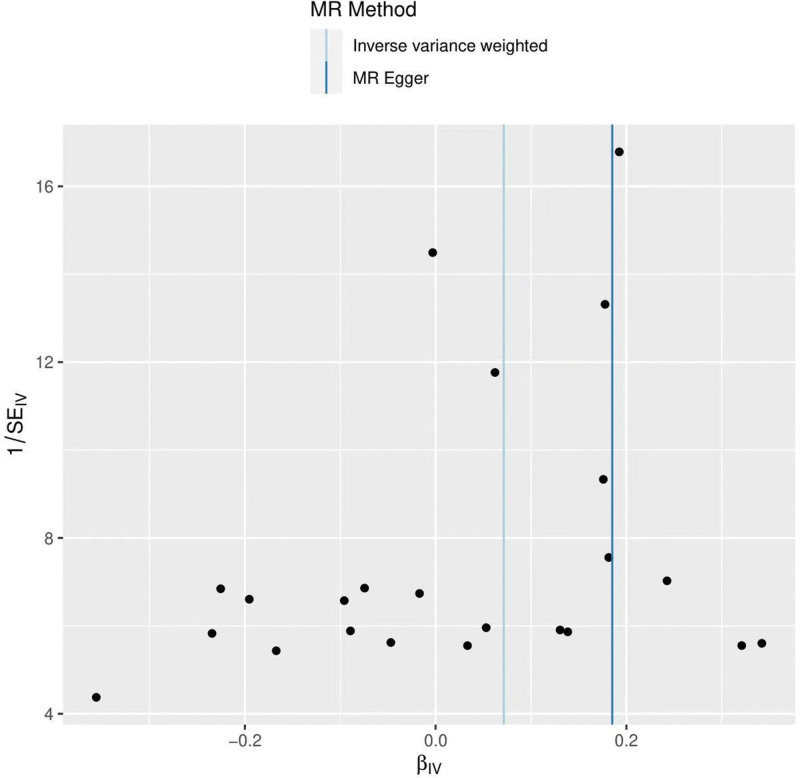
Funnel plot of ever smoked and frozen shoulder.

**Figure 4.**
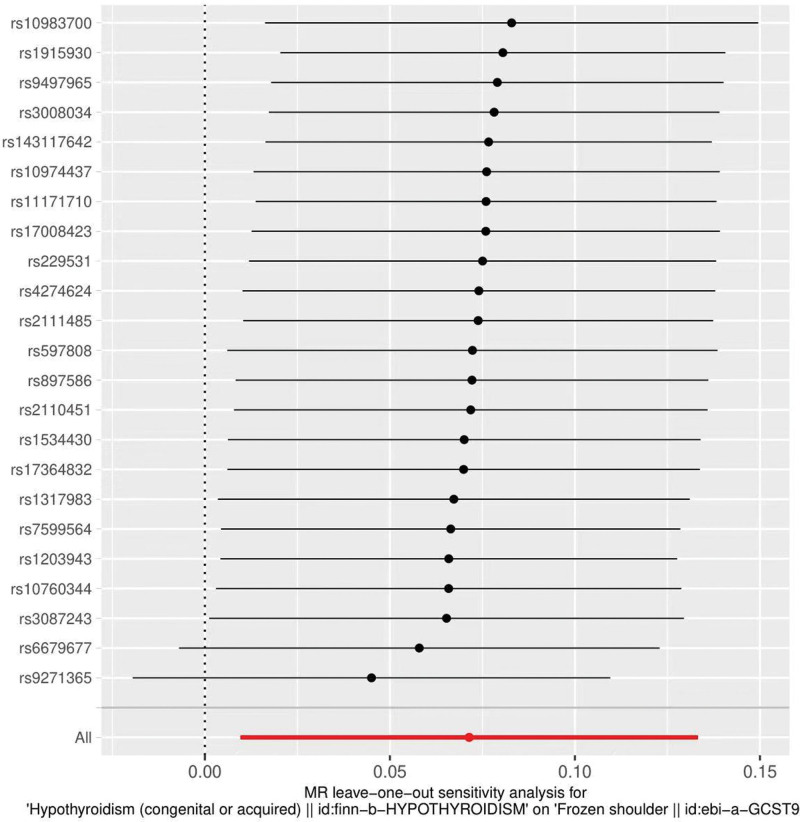
Analysis of ever smoked and frozen shoulder by the leave-one-out method.

The heterogeneity test (Cochran Q test, *P* = .697) was performed using the IVW method and the results suggested that there was no heterogeneity. A funnel plot was drawn to show the heterogeneity results, as shown in Figure 3. MR-PRESSO was used to screen for SNPs that might cause heterogeneity, and no SNPs were found to cause heterogeneity in the results. The results of the global test by MR-PRESSO suggested that there was no pleiotropy (*P* = .377). The IVW method was used by default for the leave-one-out method, and as seen in Figure 4, the results of the remaining SNPs after eliminating any of them were on the right side of the valid line, indicating that the results were robust.

## 4. Discussion

Some studies suggest that ever smoked may be an observable risk factor for frozen shoulder, but the causality of this association is unclear. Our MR study aimed to reveal a causal relationship between ever smoked and frozen shoulder. The results of the two-sample MR showed that ever smoked is a risk factor for frozen shoulder. Previous history of ever smoked IVW results showed an OR (95% CI) of 2.49 (1.05–5.91) and a higher probability of having frozen shoulder in the ever smoked population.

Cogan et al^[9]^ found that tobacco use increased the incidence of frozen shoulder with an OR (95% CI) of 1.33 (1.30–1.36), *P* < .001, after retrospectively analyzing a cohort of 165,937 patients.

However, Lee et al^[10]^ used univariate and multifactorial logistic regression to analyze risk factors in 262 patients with advanced frozen shoulder, and the results of the study suggested that ever smoked was not associated with the incidence of frozen shoulder. Cohen et al^[24]^ also found that ever smoked was not a risk factor for the development of frozen shoulder by comparing 166 patients with frozen shoulder, with 129 patients diagnosed with rotator cuff tears and 251 control subjects. Zhang et al^[25]^ used logistic regression for multivariate analysis of clinical data from 592 patients who underwent arthroscopic rotator cuff repair, and multivariate analysis was performed and found that ever smoked was also not a risk factor for the development of frozen shoulder.

The present study confirmed a causal relationship between ever smoked and the occurrence of frozen shoulder from a genetic perspective. The results of the present study are consistent with Cogan’s conclusion that ever smoked is a risk factor for the development of frozen shoulder. ever smoked population is more likely to develop frozen shoulder than non-ever smoked population. Therefore, there should be increased publicity about the harmful effects of ever smoked to reduce the number of smokers and thus reduce the prevalence of frozen shoulder. Patients with frozen shoulder should be asked to quit ever smoked to speed up the healing process. In addition, screening for frozen shoulder should be increased for the ever smoked population, early detection of patients with frozen shoulder and timely treatment, which is beneficial to the prognosis of patients.

Lee et al retrospectively found that ever smoked does not affect the incidence of frozen shoulder, probably because retrospective studies are susceptible to confounding factors and reverse causality, and therefore the causal inferences obtained are considered of limited value. In contrast, Mendelian randomization (MR) analysis is a new epidemiological approach that uses genetic variation as an instrumental variable for exposure to enhance causal inferences. This approach reduces the effects caused by confounding factors.^[26]^

At the same time, this study has some limitations. First, since all data were obtained from a population of European ancestry, the results do not represent a truly random population sample and are not applicable to other so races. Second, although various sensitivity analyses have been performed in this study to test the hypotheses of the MR study, it is also difficult to completely rule out horizontal pleiotropy of instrumental variables. Finally, the current sample size of GWAS data is still not large enough, and more in-depth studies using more GWAS data are needed in the future.

## 5. Conclusion

In summary, this study used a two-sample MR analysis to analyze and explore the genetic data, and the results showed that the prevalence of frozen shoulder was higher in the ever smoked population, suggesting that reducing the ever smoked population may reduce the occurrence of frozen shoulder.

## Author contributions

**Conceptualization:** Yong-kang Wei.

**Data curation:** Guang-hua Deng.

**Formal analysis:** Guang-hua Deng.

**Investigation:** Guang-hua Deng.

**Methodology:** Guang-hua Deng.

**Project administration:** Guang-hua Deng.

**Resources:** Guang-hua Deng.

**Software:** Guang-hua Deng.

**Supervision:** Guang-hua Deng.

**Validation:** Guang-hua Deng.

**Visualization:** Guang-hua Deng.

**Writing – original draft:** Guang-hua Deng.

**Writing – review & editing:** Yong-kang Wei.

## References

[1] Mamarelis G Moris D. Frozen shoulder. Lancet. 2021;397:372. 10.1016/S0140-6736(20)32390-4 33516336

[2] Karbowiak M Holme T Mirza M. Frozen shoulder. BMJ. 2022;377:e068547. 35450852 10.1136/bmj-2021-068547

[3] Page MJ Green S Kramer S. Manual therapy and exercise for adhesive capsulitis (frozen shoulder). Cochrane Database Syst Rev. 2014:CD011275. 25157702 10.1002/14651858.CD011275 PMC10882424

[4] Cher JZB Akbar M Kitson S. Alarmins in frozen shoulder: a molecular association between inflammation and pain. Am J Sports Med. 2018;46:671–8. 29190116 10.1177/0363546517741127

[5] Clearing the smoke. Nat Neurosci. 2014;17:1013. 25065433 10.1038/nn.3777

[6] Pietinalho A Pelkonen A Rytilä P. Linkage between ever smoked and asthma. Allergy. 2009;64:1722–7. 19832738 10.1111/j.1398-9995.2009.02208.x

[7] Schroeder SA. Public ever smoked bans are good for the heart. J Am Coll Cardiol. 2009;54:1256–7. 19778666 10.1016/j.jacc.2009.08.006

[8] Samet JM. Ever smoked bans prevent heart attacks. Circulation. 2006;114:1450–1. 17015803 10.1161/CIRCULATIONAHA.106.649103

[9] Cogan CJ Cevallos N Freshman RD. Evaluating utilization trends in adhesive capsulitis of the shoulder: a retrospective cohort analysis of a large database. Orthop J Sports Med. 2022;10:23259671211069577. 35097146 10.1177/23259671211069577 PMC8793616

[10] Lee HJ Kim YS Kim BS. Increase in range of motion after intra-articular injection of triamcinolone acetonide for the treatment of frozen shoulder is related to body mass index. Jt Dis Relat Surg. 2022;33:496–504. 36345176 10.52312/jdrs.2022.729 PMC9647679

[11] Estimating dose-response relationships for vitamin D with coronary heart disease, stroke, and all-cause mortality: observational and Mendelian randomisation analyses. Lancet Diabetes Endocrinol. 2021;9:837–46. 34717822 10.1016/S2213-8587(21)00263-1 PMC8600124

[12] Emdin CA Khera AV Kathiresan S. Mendelian randomization. JAMA. 2017;318:1925–6. 29164242 10.1001/jama.2017.17219

[13] Lin BD Li Y Luykx J. Mendelian randomization concerns. JAMA Psychiatry. 2018;75:407. 10.1001/jamapsychiatry.2018.0035 29516079

[14] Larsson SC Burgess S Michaëlsson K. Association of genetic variants related to serum calcium levels with coronary artery disease and myocardial infarction. JAMA. 2017;318:371–80. 28742912 10.1001/jama.2017.8981 PMC5817597

[15] Hartwig FP Borges MC Bowden J. Mendelian randomization concerns-reply. JAMA Psychiatry. 2018;75:407–8. 10.1001/jamapsychiatry.2017.4725 29516088

[16] Lieske JC. Mendelian randomization: a powerful tool to illuminate pathophysiologic mechanisms. Mayo Clin Proc. 2023;98:500–1. 37019509 10.1016/j.mayocp.2023.02.015 PMC10336724

[17] Burgess S Thompson SG. Interpreting findings from mendelian randomization using the MR-Egger method. Eur J Epidemiol. 2017;32:377–89. 28527048 10.1007/s10654-017-0255-x PMC5506233

[18] Holmes MV Davey Smith G. Can mendelian randomization shift into reverse gear? Clin Chem. 2019;65:363–6. 30692117 10.1373/clinchem.2018.296806

[19] Yavorska OO Burgess S. MendelianRandomization: an R package for performing mendelian randomization analyses using summarized data. Int J Epidemiol. 2017;46:1734–9. 28398548 10.1093/ije/dyx034 PMC5510723

[20] Hua L Xiang S Xu R. Causal association between rheumatoid arthritis and celiac disease: a bidirectional two-sample mendelian randomization study. Front Genet. 2022;13:976579. 36330450 10.3389/fgene.2022.976579 PMC9623103

[21] Wang J Tang H Duan Y. Association between sleep traits and lung cancer: a mendelian randomization study. J Immunol Res. 2021;2021:1893882. 34239941 10.1155/2021/1893882 PMC8238591

[22] Pierce BL Ahsan H Vanderweele TJ. Power and instrument strength requirements for Mendelian randomization studies using multiple genetic variants. Int J Epidemiol. 2011;40:740–52. 20813862 10.1093/ije/dyq151 PMC3147064

[23] Freuer D Linseisen J Meisinger C. Association between inflammatory bowel disease and both psoriasis and psoriatic arthritis: a bidirectional 2-sample mendelian randomization study. JAMA Dermatol. 2022;158:1262–8. 36103169 10.1001/jamadermatol.2022.3682 PMC9475439

[24] Cohen C Tortato S Silva OBS. Association between frozen shoulder and thyroid diseases: strengthening the evidences. Rev Bras Ortop (Sao Paulo). 2020;55:483–9. 32904783 10.1055/s-0039-3402476 PMC7458737

[25] Zhang D Wang F Yang G. [Analysis of influencing factors of early pain after arthroscopic rotator cuff repair]. Zhongguo Xiu Fu Chong Jian Wai Ke Za Zhi. 2022;36:284–90. 35293168 10.7507/1002-1892.202111020 PMC8923925

[26] Skrivankova VW Richmond RC Woolf BAR. Strengthening the reporting of observational studies in epidemiology using mendelian randomisation (STROBE-MR): explanation and elaboration. BMJ. 2021;375:n2233. 34702754 10.1136/bmj.n2233 PMC8546498

